# *In Situ* Reduced Graphene Oxide and Polyvinyl Alcohol Nanocomposites With Enhanced Multiple Properties

**DOI:** 10.3389/fchem.2022.856556

**Published:** 2022-03-22

**Authors:** Wenwen Hu, Shuhan Liu, Zhonghai Wang, Xianjing Feng, Ming Gao, Fangming Song

**Affiliations:** ^1^ Guangxi Engineering Center in Biomedical Materials for Tissue and Organ Regeneration, the First Affiliated Hospital of Guangxi Medical University, Nanning, China; ^2^ Guangxi Collaborative Innovation Center of Regenerative Medicine and Medical Bioresource Development and Application, the First Affiliated Hospital of Guangxi Medical University, Nanning, China; ^3^ Information and Management College, Guangxi Medical University, Nanning, China; ^4^ Pharmaceutical College, Guangxi Medical University, Nanning, China

**Keywords:** graphene oxide1, polyvinyl alcohol, nanocomposites, *in situ* reduction, electrical conductivity

## Abstract

The nanocomposites formed by graphene oxide (GO) and carbazate-modified polyvinyl alcohol (PVA-N) were developed to investigate their multiple properties for wide applications. Their physicochemical characterizations confirmed that the *in situ* reduced GO (rGO) not only decreased the crystallization but also induced the porous structures inside the nanocomposites. Significantly, it revealed that the comprehensive performance of PVA-N2-2%GO consisted of PVA-N2 with the carbazate degree of substitution (DS) of 7% and the weight ratio (wt%) of 2% GO displayed 79% of tensile elongation and tensile strength of 5.96 N/mm^2^ (MPa) by tensile testing, glass transition temperature (Tg) of 60.8°C and decomposition temperature (Td) of 303.5°C by TGA and DSC, surface contact angle at 89.4 ± 2.1°, and electrical conductivity of 9.95 × 10^−11^ S/cm. The abovementioned comprehensive performance was enhanced with the increased amount of *in situ* rGO, contributed by the high DS of the carbazate group in PVA-N and high amount of GO. The rGO by *in situ* reduction was the main driving force for enhancing the multiple properties inside the nanocomposites.

## Introduction

Due to their fascinating properties, graphene oxide (GO) and its derivatives including the reduced GO (rGO) and functionalized graphene have been widely applied in various fields such as sensors (Xu and Zhang, 2017), energy devices (Cai and Yu, 2019), membranes (Zhu et al., 2020; Cheon et al., 2021), biomedicines (Liu et al., 2021; Yan et al., 2022), coatings (Kulyk et al., 2021), and polymer composites (Li et al., 2018). Furthermore, GO and its derivatives have extremely high tensile and compressive strength, but in “solid state,” these properties are not prominently observed because of the fact that the compressed and random surface flaws always cause them to crack and fail (Chabot et al., 2014). On the other hand, polymers such as epoxies, polystyrene, polyvinyl alcohol (PVA), and polyesters are limitedly applied in many fields, attributed to the lack of mechanical, thermal, or electrical properties (Balazs et al., 2006). Therefore, the novel nanocomposites have attracted tremendous attention by combining the merits of GO or its derivatives with different types of polymer matrices (Yusuf et al., 2019; Zhang et al., 2019). Since the final polymer nanocomposites are combined by a polymer system plus GO or its derivatives, their overall properties will combine some properties of the polymer on its own as well as that of GO or its derivatives on their own (Bayan and Karak, 2017). On this account, the addition of 1 wt% GO to the epoxy resins had reached similar effect on improving the thermal conductivity with that of the epoxy resins after the incorporation of 1 wt% of single wall carbon nanotubes. Meanwhile, 5 wt% GO-epoxy composites displayed a thermal conductivity of around 1 W/mK, four times higher than that of the neat epoxy resin (Wang et al., 2009). It was also reported that the thermal conductivity could be increased to around 6.44 W/mK by 20 wt% GO (Guo et al., 2017). In addition, a composite thin film consisting of functionalized graphene sheets (FGS) such as the filler and polystyrene (PS) as the host material exhibited conductivity values ranging from 1 to 24 s/m with the change of FGS portions (Yu et al., 2008).

Generally, to obtain the remarkable enhancement of overall performance, it always anticipated that GO and its derivatives achieved a good dispersion and had a strong interface interaction inside the polymer matrix (Ponnamma et al., 2021). As a water-soluble, biocompatible, and nontoxic polymer, PVA has been considered an ideal polymer matrix to prepare composites owing to the abundance of hydroxyl groups, which makes the GO-type nanomaterials uniformly dispersed (Tan et al., 2015). Thus, GO was exfoliated and nano-dispersed within the PVA matrix, and the final nanocomposites possessed high mechanical and thermal properties and increased the barrier properties with up to 1 wt% GO (Morimune et al., 2012). In the meantime, the PVA nanocomposite containing 3 wt% GO displayed 4.8 GPa in Young’s modulus and 110 ± 7 MPa in tensile yield strength as well as 36 ± 4% elongation (Xu et al., 2009). Significantly, appropriate ultrasonication could easily produce defects on the surface of GO and reach effective load transfer between the PVA matrix and GO to obtain remarkable mechanical strength (Qi et al., 2013).In addition, it also indicated that PVA-GO (1: 1) nanocomposites after borate treatment behaved 360 MPa in tensile strength and fourfold enhancement in toughness compared with nacre (Liu et al., 2013). Evidently, the incorporation of GO and its derivatives enhances the mechanical and thermal properties of PVA nanocomposites.

Although the incorporation of GO and its derivatives could provide enhanced mechanical and thermal properties, it was disappointing that improved electrical conductivity was rarely observed because of the originality of GO, with certain oxygen-containing functional groups including hydroxyls, carboxyls, carbonyls, and epoxides (Ponnamma et al., 2021). However, graphene or rGO was directly applied to form nanocomposites with PVA to improve their electrical conductivity in parallel with mechanical and thermal properties (Jiang et al., 2010). Unfortunately, graphene or rGO with a high specific surface area tends to form irreversible agglomerates, which leads to poor dispersion and weak interface interaction inside the polymer matrix (Mondal et al., 2021). To make up the abovementioned drawbacks, the nanocomposites were initially prepared by mixing PVA and GO followed by hydrazine or ascorbic acid reduction (Ma et al., 2015). The resulting nanocomposites revealed hydrophobicity with surface contact angle above 90° and possessed dramatically enhanced electrical conductivity in contrast to PVA or PVA-GO without reduction (Ma et al., 2016). Nevertheless, the limitation always existed due to the fact that post reduction still happened under aqueous condition which affected the effective dispersion of rGO.

Herein, the carbazate modified PVA (PVA-N) was prepared and mixed with GO to form nanocomposites after thermal treatment. The rGO was formed inside the nanocomposites by the *in situ* reduction of carbazate groups of PVA-N. And the resulting nanocomposites were characterized and confirmed their enhanced thermal property, mechanical behavior, hydrophobic ability, and electrical conductivity. Significantly, the mechanism of enhanced multiple properties was also confirmed to be driven by *in situ* rGO inside the nanocomposites.

## Experimental

### Materials

Polyvinyl alcohol (PVA, Mw = 47,000, 98.0–98.8 mol% hydrolysis), graphene oxide solution (2 mg/ml, dispersion in H_2_O), graphene oxide powder (15–20 sheets, 4–10% edge-oxidized), dimethyl sulfoxide (DMSO, ≥ 99.9%), 1, 1′-carbonyldiimidazole (CDI, ≥ 97.0%), hydrazine hydrate (80%, Sigma), sodium tetraborate decahydrate (Na_2_B_4_O_7_, ≥99.5%, Sigma), tert-butyl carbazate (≥ 98.0%, Sigma) and 2, 4, 6-trinitrobenzene sulfonic acid [TNBS, 5% (w/v) in H_2_O] were purchased from Sigma Aldrich and directly used without further purification.

### Preparation of PVA-N

PVA of 1 g was added to 20 ml DMSO, and the mixture was heated at 90°C for 15 min till PVA was totally dissolved. After cooling at room temperature, 2 g CDI was added and the solution was stirred for 24 h. Later, 2.1 or 4.2 ml of hydrazine hydrate was added, which was left to stir for another 24 h. Finally, the reaction mixture was diluted with water and dialyzed (Spectra/Por^®^6 Dialysis Membrane, MWCO: 3.5 kD) for 3 days, and the PVA-N was isolated as white powder after lyophilization. The synthetic steps are illustrated in Supplementary Figure S1, and the detailed components of PVA-N preparation are listed in Supplementary Table S1.

### Preparation of PVA Nanocomposites

The PVA nanocomposites were prepared by simple thermal treatment. Details are as follows: PVA or PVA-N was dissolved in deionized (DI) water at 90°C to reach a totally soluble (20 mg/ml) state. Then, the defined volume of GO suspension (2 mg/ml) was dripped into the abovementioned solution with sonication for 1 h to make sure that GO was fully dispersed inside the solution. Finally, the mixtures were cast in a cut-off of 25 ml syringe, initially dried at 40°C for 2 days and further heated at 80°C for another 2 days. The nanocomposite films were peeled off from the syringe for further experiments. Specifically, for mechanical testing, the mixtures were cast in a rectangle teflon substrate (30 mm length× 15 mm width) before thermal treatment. The detailed recipe for the preparation of nanocomposites and their corresponding names are listed in Supplementary Table S2.

### Characterization

The DS of carbazate groups in PVA-N was investigated by TNBS assay as previously described (Yang et al., 2020). Details are as follows: samples of 1 mg each were dissolved in 20 ml Na_2_B_4_O_7_ buffer (pH = 9.3, 0.1 M). Then, 1 ml sample solution was mixed with 25 μL TNBS solution for further testing. After 3 h reaction, the mixture was analyzed by UV-vis spectroscopy (UV-2600/2700, Shimadzu) at 505 nm and compared to a standard curve based on tert-butyl carbazate. The PVA and PVA-N were additionally characterized by ^1^H and ^13^C nuclear magnetic resonance (NMR, Jeol JNM-ECP Series FT NMR) at 40°C.

The molecular structure of PVA, PVA-N, and their nanocomposites was also characterized by Fourier transform infrared spectra (FTIR, PerkinElmer spectrum One FT-IR spectrometer), X-ray photoelectron spectroscopy (XPS, Physical Systems Quantum 2000 spectrometer) with monochromatic Al Kα radiation, X-ray diffraction (XRD, Diffraktometer D5000, Siemens, Germany) with Bragg–Brentano geometry and Cu Kα radiation (*λ* = 1.54 Å) and diffraction angle from 3 to 50°, and Raman spectroscopy (Renishaw inVia Raman spectrometer) equipped with a Leica LM optical microscope and an argon ion laser (*λ* = 514.5 nm) source.

The thermal properties of PVA, PVA-N, and their nanocomposites were investigated by thermogravimetric analysis (TGA, TA instruments TGAQ500) with the heating speed of 5°C/min from room temperature to 600°C under N_2_ atmosphere and differential scanning calorimetry (DSC, TA Instruments DSC Q1000) with the heating flow of 10°C/min from room temperature to 200°C.

The mechanical properties of the film materials were evaluated by a tensile machine (Shimadzu Autograph AGS-X) using a deformation rate of 1 mm/min. After stretch testing, the cross-section of the film materials was also investigated by scanning electron microscopy (SEM, Leo 1550 SEM instrument Zeiss, Germany) with an accelerating voltage of 5 kV after gold coating.

To study the hydrophilic and hydrophobic capacity, the film materials were fixed on the platform by double tapes and analyzed by a video-based optical contact angle measuring system (OCA15EC, dataphysics) with the droplet of 2.5 μL/s.

The body electrical conductivity was measured by a high-resistance meter (4339B, Keysight Technologies), and each samples were implemented for three parallel runs.

## Results and Discussion

**FIGURE 1:**
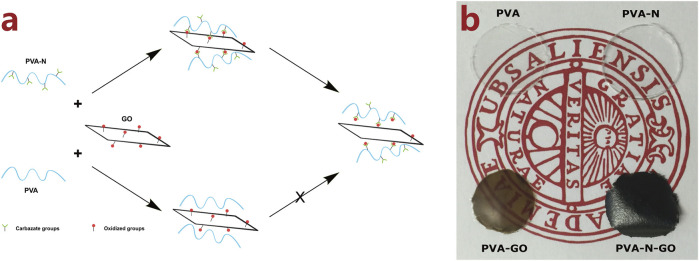
Schematic illustration of the preparation of PVA and PVA-N nanocomposites with GO after thermal treatment **(A)**, and the picture of PVA, PVA-N, PVA-GO, and PVA-N-GO with the logo of Uppsala University **(B)**.

The -OH groups of PVA were initially activated by CDI followed by reacting with hydrazine to obtain PVA-N. The PVA or PVA-N solution was mixed with different defined amounts of GO solution to form nanocomposites after thermal treatment. Figure 1A schematically illustrates the preparation of PVA and PVA-N nanocomposites, and the possible resulting chemical composition after thermal treatment. Simultaneously, the resulting nanocomposite films PVA, PVA-N, PVA-GO, and PVA-N-GO were separately optical-imaged with the background logo of Uppsala University. As shown in Figure 1B, PVA and PVA-N were transparent, while the color of PVA-GO became brown, similar to that of GO solution itself. However, the color of PVA-N-GO became black. The brown color of PVA-GO was attributed to the incorporation of GO, while the obvious dark color appeared for PVA-N-GO because of the formation of *in situ* rGO inside the nanocomposite.

### Structure and Morphology

The DS of the carbazate groups in PVA-N was obtained by TNBS assay. Two different PVA-N were defined respectively as: PVA-N1 (DS = 4%) and PVA-N2 (DS = 7%).In addition, ^1^H and ^13^C NMR were performed to characterize PVA-N. Compared to the pristine PVA, the chemical shift observed at 5.25 ppm corresponded to the protons of the amino groups for PVA-N (Supplementary Figure S2). Additionally, as indicated in Supplementary Figure S3, the characteristic C of PVA was observed at 43 ppm and 65–68 ppm for the chemical shift of ^13^C NMR. Meanwhile, the appearance of chemical shift at 160 ppm (-C=O) also proved the formation of PVA-N. In the meantime, the PVA, PVA-N, and their nanocomposites were also characterized by FTIR. As shown in Supplementary Figure S4, the apparent peak observed at 1,698 cm^−1^ provided the proof of the existence of ester groups in PVA-N and their nanocomposites with GO. From the abovementioned details, the successful modification of carbazate groups for PVA-N is confirmed.

**FIGURE 2:**
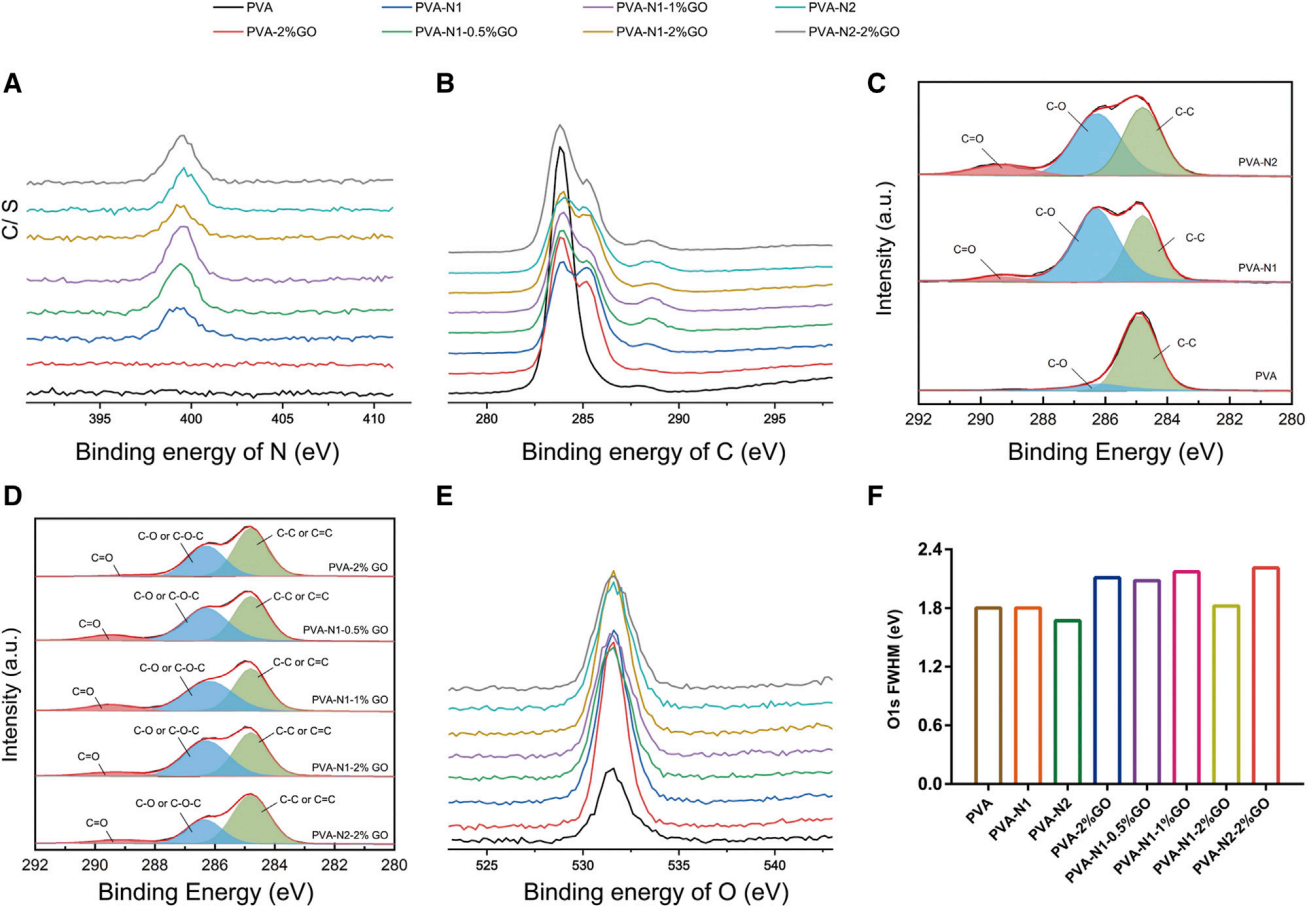
Detailed XPS spectrums of PVA, PVA-N, and their nanocomposites: N1s **(A)**, C1s **(B),** and its peak fitting **(C,D)**, and O1s **(E)** and its full width at half maxima (FWHM) **(F)**.

Furthermore, XPS was applied to characterize PVA, PVA-N, and their nanocomposites with GO. The full spectrums of samples are illustrated in Supplementary Figure S5. It was evidently observed that the N elements existed at 399 eV for PVA-N1, PVA-N2, and their corresponding nanocomposites compared with PVA and its nanocomposites, PVA-2%GO, also indicated the successful preparation of PVA-N. The existence of N elements was also clearly observed for a detailed N1s spectrum for PVA-N and its nanocomposites (Figure 2A). As shown in Figure 2B, the pristine PVA only showed a simple peak at 284 eV, while the overlapped peaks were presented for PVA-2%GO due to the contribution of oxidized groups of GO for C1s spectrum. Similarly, the obvious overlapped peaks were also observed for PVA-N and its nanocomposites, resulting from the carbazate groups of PVA-N and also the oxidized groups of GO. The peak fitting of C1s is displayed in Figures 2C,D. The three separated peaks at 289.3, 286.2, and 284.8 eV, corresponding to C=O, C-O, and C-C of PVA-N, respectively, were evidently observed for PVA-N compared to PVA with a big peak at 286.2 eV plus a relative small peak at 289.3 eV (Figure 2C). The peak at around 289.3 eV was attributed to the carbazate groups of PVA-N and its intensity was enhanced with the increase of DS of the carbazate groups. Conversely, the intensity of the peak at around 286.2 eV increased and small peak at around 289.3 eV was observed for PVA-2%GO compared to PVA alone. The enhanced intensity of the peak (C-O) and the new existed peak (C=O) for PVA-2%GO was attributed to GO itself. Meanwhile, the observed peak at around 289.3 eV also existed for PVA-N-GO, which decreased with the increase in GO wt% or high DS of the carbazate groups. For PVA-N1-GO, increasing the GO wt% induced more existed rGO, leading to the decrease of oxidized groups of GO in the nanocomposites as well as PVA-N2 with a higher DS also contributed to more rGO. However, no new peaks were evidently observed for O1s spectrum (Figure 2E). From the full width at half maxima (FWHM) of O1s, it was 1.80, 1.80, and 1.67 for PVA, PVA-N1, and PVA-N2, respectively, while it became 2.11, 1.82, and 2.21, respectively, after 2% GO incorporation. If increasing the GO wt% from 0.5%, 1%–2%, the FWHM was 2.08, 2.17, and 1.82, respectively (Figure 2F). The incorporation of GO increased the FWHM of nanocomposites, indicating more oxygen containing groups inside. However, the FWHM decreased with the increase of GO wt% and also high DS of the carbazate groups for PVA-N-GO, attributed to more rGO existed inside the corresponding nanocomposites (Rahoui et al., 2018; Zhang et al., 2022).

**FIGURE 3:**
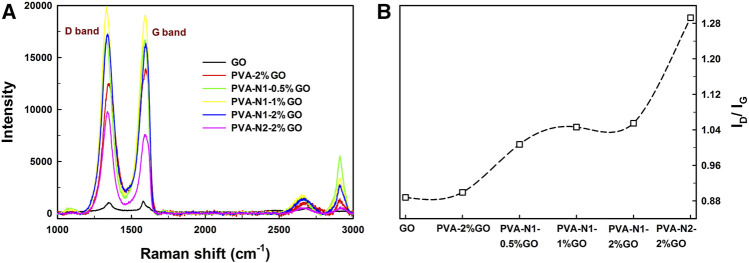
Raman spectroscopy curves **(A)** and plotted I_D_/I_G_ ratios **(B)** of GO and nanocomposites.

To further characterize the formation of rGO in PVA-N-GO, Raman spectroscopy was applied. As shown in Supplementary Figure S6, PVA and PVA-N provided their specific fingerprints at 1,000–1,500 and 2,800–3,000 cm^−1^ , respectively, and no obvious changes of Raman shifts were observed before and after the carbazate modification except for their relative intensity. Conversely, for GO alone, the D bands and G bands were observed at 1,330 and 1,595 cm^−1^ , respectively, and 2D bands also appeared at 2,669 cm^−1^. After forming the nanocomposites with PVA, their D bands and G bands presented evidently enhanced intensity as well as the specific fingerprints of PVA also retained (Figure 3A). The only difference observed was the relative intensity ratio (I_D_/I_G_) for the different nanocomposites. Therefore, the relative intensity ratios are illustrated in Figure 3B. The I_D_/I_G_ ratio was around 0.89 and 0.90 for GO alone and PVA-2%GO, respectively. Significantly, the ratio increased to 1.05 and 1.29 for PVA-N1-2%GO and PVA-N2-2%GO, respectively. In addition, increasing the GO amount also contributed to high I_D_/I_G_ ratio, which increased from 1.02 to 1.05 with the increase of GO amount from 0.5 to 2 wt%. Previous work reported that the high I_D_/I_G_ ratio was attributed to more rGO existed (Qi et al., 2019). Therefore, the higher DS of the carbazate groups and higher amount of GO induced the formation of more rGO inside the nanocomposites.

**FIGURE 4:**
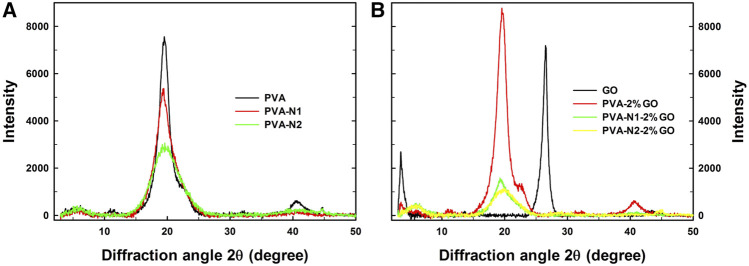
XRD curves of PVA, PVA-N1, and PVA-N2 **(A)**, and their nanocomposites with 2 wt% GO **(B)** with the diffraction angle from 3 to 50°.

The crystallization of PVA, PVA-N, and their nanocomposites with GO was additionally investigated by XRD with the diffraction angle from 3 to 50°. The pristine PVA possessed apparent diffraction angles at 19.5° and 40.7° while PVA-N1 and PVA-N2 presented the same diffraction angles except for weakened intensity (Figure 4A). The carbazate modification affected the crystallization of PVA, and the higher DS of the carbazate groups induced the weaker intensity of diffraction angles. In addition, as shown in Figure 4B, GO alone had the diffraction angles at 3.5° and 26.5°, respectively. However, the diffraction angle at 26.5° disappeared after forming PVA-2%GO. The new diffraction angle appeared at 19.5°, contributed by the PVA matrix (Bulla et al., 2021). Meanwhile, the diffraction angle observed at 3.5° also existed for PVA-2%GO, indicating that GO maintained its original state inside the nanocomposites. Conversely, compared to PVA-2%GO, the diffraction angle was vanished at 3.5°, and declined intensity was evidently observed at 19.5° and 40.7° for PVA-N1-2%GO and PVA-N2-2%GO, respectively, indicating that the *in situ* rGO was well dispersed inside the nanocomposites (Teo et al., 2012). Most importantly, increasing the amount of GO led to the decreased intensity at 19.5° for PVA-N1-GO nanocomposites (Supplementary Figure S7). From the abovementioned details, the crystallization degree reduced after forming the nanocomposites (Malas et al., 2017). In particular, for PVA-N-GO, the rGO was well dispersed in the PVA-N matrix, leading to weaken crystallization.

**FIGURE 5:**
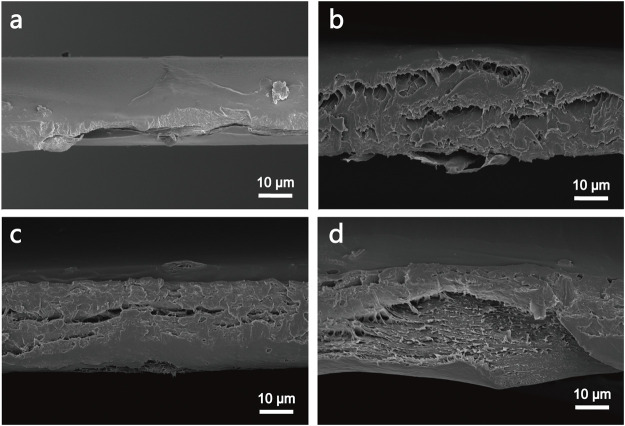
SEM images of the cross-section of PVA **(A)**, PVA-N1 **(B)**, PVA-2%GO **(C)**, and PVA-N1-2%GO **(D)**.

The morphology of cross-section of PVA, PVA-N, and their nanocomposites was investigated by SEM. As illustrated in Figure 5, the simple PVA exhibited the dense packed structures. However, the layered structures were observed for PVA-2%GO and PVA-N1 (Figures 5B,C). In particular, some porous structures were observed for PVA-N1-2%GO. The formation of nanocomposites could loosen the dense structures and improve their permeability (Yang et al., 2016).

### Thermal Behaviors

**FIGURE 6:**
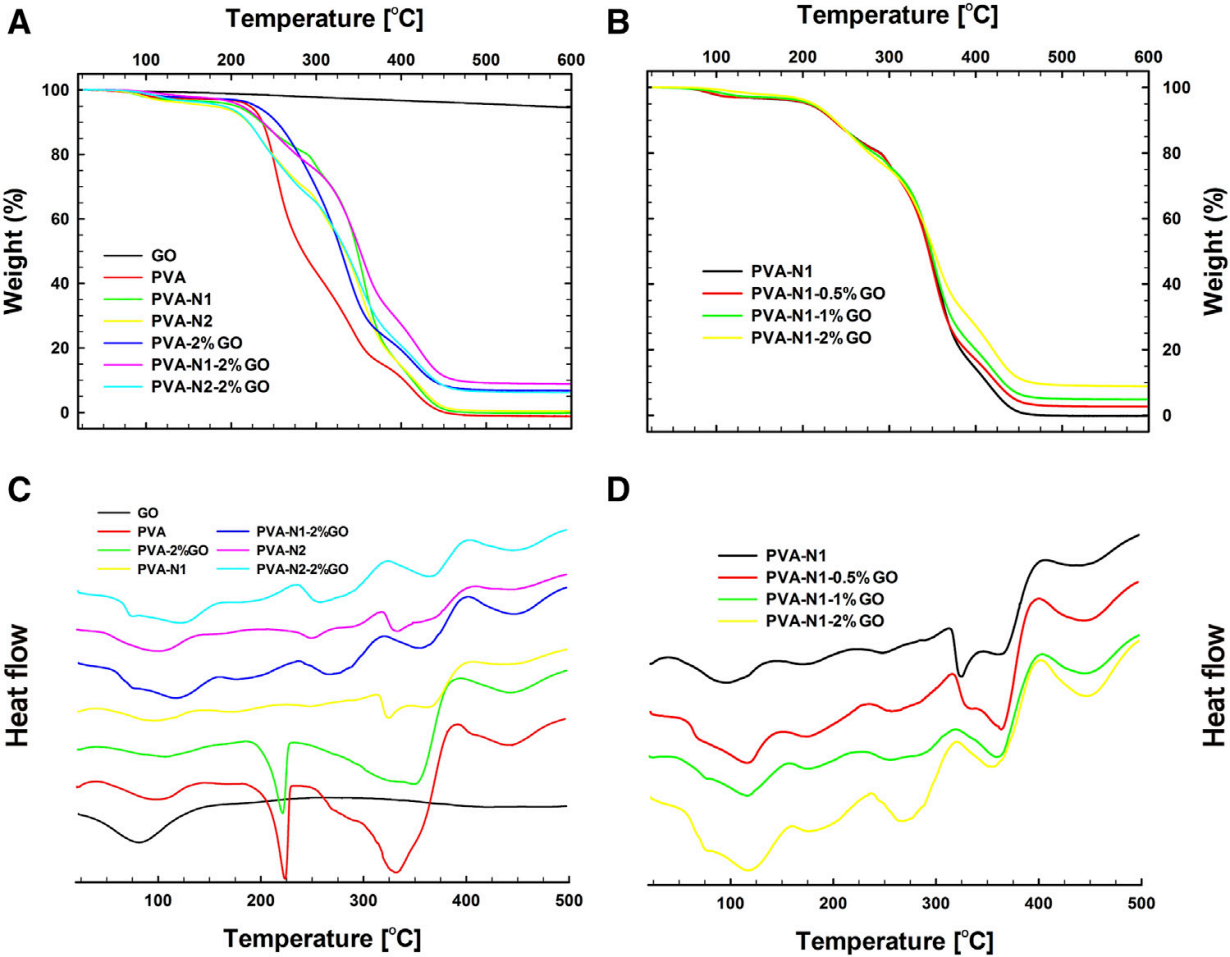
Thermal behaviors of PVA, PVA-N, and their nanocomposites. TGA **(A)** and DSC **(C)** results of GO, PVA, PVA-N1, PVA-N2, PVA-2%GO, PVA-N1-2%, and PVA-N2-2%GO, and TGA **(B)** and DSC **(D)** results of PVA-N1 and its nanocomposites: PVA-N1-0.5%GO, PVA-N1-1%GO, and PVA-N1-2%GO.

Thermal behavior is one of the important parameters for films in practical application. The thermal behaviors of nanocomposites were investigated by TGA and DSC, respectively. As shown in Figure 6A, GO alone only presented 5% of weight loss, while the pristine PVA had 100% of weight loss from 26 to 600°C. Similarly, PVA-N also exhibited 100% of weight loss during the temperature ranges. Nevertheless, around 10% weight was saved for PVA-2%GO, PVA-N1-2%-GO, and PVA-N2-2%GO, due to the existence of GO inside the nanocomposites (Figure 6A). In addition, as shown in Figure 6B, it exhibited the TGA results of PVA-N1 with different wt% of GO. It was observed that increasing the amount of GO could decrease the weight loss of resulting nanocomposites. And 5% of weight loss was observed for GO due to the oxidized groups of GO, while the decreased weight loss of nanocomposites resulted from the incorporation of GO. The corresponding Td’s from TGA results are listed in Table 1. It was 235.2°C for PVA, while it increased to 269.5°C for PVA-2%GO with the corporation of GO. Conversely, the Td was 301.2 and 303.5°C for PVA-N1-2%GO and PVA-N2-2%GO, respectively, which increased separately compared to that of PVA-N1 and PVA-N2 (272.9 and 282.1°C). And increasing the wt% of GO from 0.5, 1, and 2%, the corresponding Td became 295.4, 299.7, and 301.2°C, respectively. The Td of PVA mainly depended on the -OH groups and the formed hydrogen bonds. Although the carbazate groups of PVA-N reduced the hydrogen bonds of -OH groups, the defined physical barrier effect and the newly formed hydrogen bonds in PVA-N resulted in the enhanced thermal stability (Ali et al., 2011; Zhou et al., 2012; Manna et al., 2016). Additionally, the introduction of GO formed another physical barrier effect and led to the higher Td of the nanocomposites. A slight increase in Td of PVA-N-2%GO compared to PVA-2%GO was determined by the rGO formed hydrogen bonds inside the nanocomposites.

**TABLE 1:** Properties of PVA, PVA-N, and their nanocomposites with GO.

Name	Decomposition temperature (°C)	Tg (°C)^a^	Tensile elongation (%)^b^	Tensile strength (MPa)^b^	Young’s modulus (MPa)^b^
PVA	235.2	47.7	124	14.07	2782.60
PVA-2%GO	269.5	50.1	42	4.43	—
PVA-N1	272.9	50.8	231	12.39	2876.33
PVA-N1-0.5%GO	295.4	53.6	116	9.49	2017.44
PVA-N1-1%GO	299.7	54.5	112	6.78	1153.53
PVA-N1-2%GO	301.2	56.9	80	5.03	—
PVA-N2	282.1	48.2	170	12.29	3003.66
PVA-N2-2%GO	303.5	60.8	79	5.96	—

a means data obtained from TGA and DSC in Figure 6.

b means data obtained from stress-strain measurements in Supplementary Figure S8.

The DSC results of PVA, PVA-N, and their nanocomposites were displayed in Figures 6C,D. As indicated in Figure 6C, GO only displayed an apparent glass transition at 43.1°C. However, PVA and its nanocomposites presented a clear decomposition transition at 180–225°C, while it was not evidently observed for PVA-N1, PVA-N2, and their nanocomposites. No obvious differences in the heat flux versus temperature were observed for PVA-N1 with different wt% of GO except that the transition became more evidently observed with the increase of GO wt% (Figure 6D). Table 1 also exhibits the Tg of PVA, PVA-N, and their nanocomposites. The Tg of PVA was 47.7°C, which increased to 50.8 and 48.2°C for PVA-N1 and PVA-N2, respectively, after carbazate modification. Meanwhile, the Tg became 50.1°C for PVA-2%GO, while it was 56.9°C for PVA-N1-2%GO and 60.8°C for PVA-N2-2%GO. Similarly, increasing the GO amount also enhanced the Tg of final nanocomposites, which was 53.6, 54.5, and 56.9°C for PVA-N1 with 0.5%, 1%, and 2% GO, respectively. Hydrogen bonds played the main role in Tg of the abovementioned materials (Wang et al., 2021). Therefore, the newly formed hydrogen bonds between -OH groups of PVA and oxidized groups of GO, and -NHNH_2_ and -OH groups of PVA-N contributed to the increased Tg of PVA-GO and PVA-N comparing with pristine PVA (Moulay, 2015). Furthermore, the growing Tg in PVA-N-GO mainly depended on the incorporation of rGO inside the nanocomposites.

### Mechanical Properties

Tensile testing was carried out to evaluate the mechanical properties of samples withstanding axial loading. Supplementary Figure S8 illustrates the stress-strain curves of PVA, PVA-N, and their nanocomposites with GO. After further calculation, their tensile elongation, tensile strength, and Young’s modulus are respectively listed in Table 1. The tensile elongation for pristine PVA was 124% and it became 231% and 170% for PVA-N1 and PVA-N2, respectively, in contrary to tensile strength which slightly decreased after carbazate modification. And the tensile elongation and strength significantly reduced after incorporation of GO, where they were 42% and 4.43 MPa for PVA-2%GO, 80% and 5.03 MPa for PVA-N1-2%GO, and 79% and 5.96 MPa for PVA-N2-2%GO. However, PVA-N displayed a higher tensile elongation and strength than those of PVA after the incorporation with the same amount of GO. Similarly, PVA-N behaved better Young’s modulus than that of PVA. However, the incorporation of GO led to the decrease of Young’s modulus. Significantly, PVA and PVA-N with 2% GO were above the calculation limits so that it was impossible to obtain their Young’s modulus. The carbazate modification and GO incorporation both reduced the crystallization of the pristine PVA. Although a certain hydrogen bonding vanished, the carbazate groups also offered newly formed hydrogen bonding for PVA-N. In parallel, incorporation of GO not only reduced the hydrogen bonding inside the PVA, but also created the newly formed hydrogen bonding between the oxidized groups and -OH or carbazate groups. Accordingly, the carbazate groups donated the formation of newly formed hydrogen bonding and the decrease of crystallization, which induced high tensile elongation and Young’s modulus than those of PVA alone. The *in situ* rGO also contributed more favorable mechanical behaviors than those of GO after forming the nanocomposites (Gholampour et al., 2017).

### Hydrophilicity and Hydrophobicity

**FIGURE 7:**
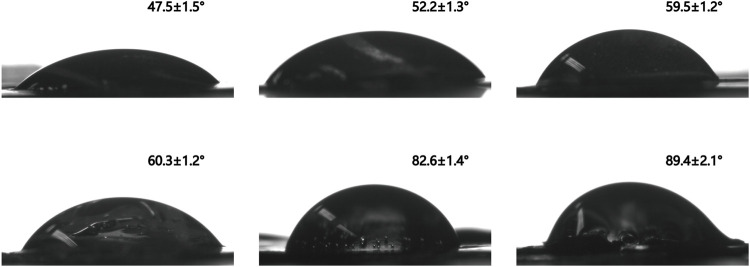
Surface contact angle images of PVA **(A)**, PVA-N1 **(B)**, PVA-N2 **(C)**, PVA-2%GO **(D)**, PVA-N1-2%GO **(E)**, and PVA-N2-2%GO **(F)**.

The hydrophilicity and hydrophobicity of PVA, PVA-N, and their nanocomposites was implemented by the surface contact angle with a certain droplet speed. After the carbazate modification, the surface contact angle switched from 47.5 ± 1.5° for PVA to 52.2 ± 1.3° and 59.5 ± 1.5° for PVA-N1 and PVA-N2, respectively (Figure 7). Significantly, the formation of nanocomposites also led to the increase of the surface contact angle. It became 71.5 ± 1.8°, 79.9 ± 0.5°, and 82.6 ± 1.4° for PVA-N1 separately mixing with 0.5%, 1%, and 2% GO, respectively (Figure 7; Supplementary Figure S9). Furthermore, the surface contact angle also grew to 89.4 ± 2.1° for PVA-N2-2%GO compared to 60.3 ± 1.2° for PVA-2%GO (Figure 7). The slight increase of the surface contact angle for PVA-N1 and PVA-N2 resulted from the carbazate groups’ modification, leading to a reduced hydrogen bonding inside the PVA-N. The surface contact angle increased for PVA-N-2%GO compared to PVA-2%GO after being incorporated with the same amount of GO due to the *in situ* rGO, which contributed to the hydrophobicity inside the nanocomposites (Etmimi et al., 2013).

### Electrical Conductivity

**FIGURE 8:**
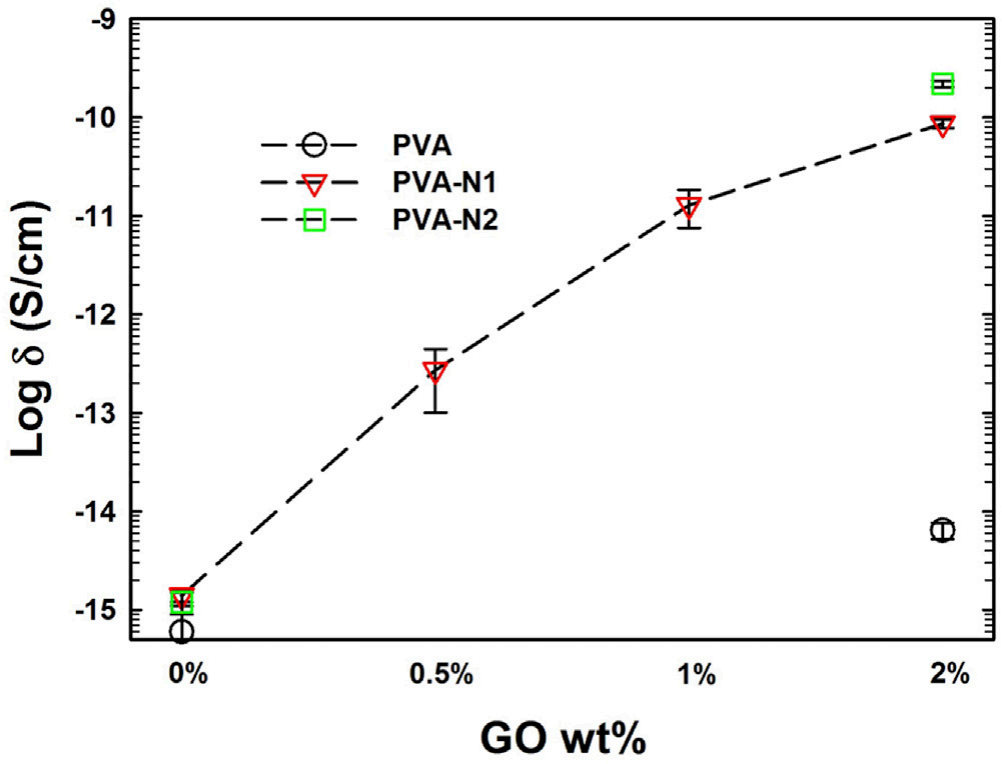
Electrical conductivity of PVA, PVA-N, and their nanocomposites.

Due to its natural conductivity, graphene was always considered to be applied to improve the electrical conductivity of nanocomposites or matrices. The electrical conductivity of PVA and PVA-N maintained at 10^−15^ levels before and after modification (Figure 8). Significantly, the conductivity was enhanced after the incorporation of GO and increased with the increase of the amount of GO inside the nanocomposites. However, the enhanced electrical conductivity of PVA-N-GO was around 10^3^ times more than that of PVA-GO after incorporating 2% GO. The poor conductivity for PVA-GO was attributed to the oxidized groups of GO, which led to the formation of disrupted conjugation and lattice defects (Mo et al., 2015). On the contrary, the *in situ* rGO contributed to the enhanced electrical conductivity for PVA-N-GO.

## Conclusion

The novel nanocomposites consisted of PVA-N incorporated with GO after heat treatment were developed to investigate their enhanced multiple properties. PVA-N-GO possessed loose structures, decreased crystallization degree, and increased surface contact angle after GO incorporation. Meanwhile, the newly formed PVA-N-GO displayed remarkable enhancements in thermal properties, mechanical behaviors, and electrical conductivity, and small improvements in hydrophobicity compared to PVA-GO. Significantly, the high DS of PVA-N and high amount of GO induced high improvement of the overall properties of PVA-N-GO. Crystallinity, hydrogen bonding among PVA-N-GO, and physical barriers from the incorporated rGO are the main reasons for affecting the structures and properties. The *in situ* rGO induced by PVA-N is the central reason for multiple properties enhancements in the PVA-N-GO.

## Data Availability

The original contributions presented in the study are included in the article/Supplementary Material, further inquiries can be directed to the corresponding authors.
