# Supplementary material for: In Situ Reduced Graphene Oxide and Polyvinyl Alcohol Nanocomposites With Enhanced Multiple Properties
Source: Front Chem. 2022 Mar 22;10:856556. doi: 10.3389/fchem.2022.856556 (PMC8980314; doi:10.3389/fchem.2022.856556)
Supplement: Supplementary file 1 [file DataSheet1.docx]

In situ reduced graphene oxide and polyvinyl alcohol nanocomposites with enhanced multiple properties

Wenwen Hu ^1, 2 #^, Shuhan Liu ^1, 2 #^, Zhonghai Wang ^3 #^ , Xianjing Feng ^1,^ ^4^, Ming Gao ^1, 2 *^, and Fangming Song ^1, 2 *^

^1^ Guangxi Engineering Center in Biomedical Materials for Tissue and Organ Regeneration, The First Affiliated Hospital of Guangxi Medical University, Nanning, 530021, China

^2^ Guangxi Collaborative Innovation Center of Regenerative Medicine and Medical Bioresource Development and Application, The First Affiliated Hospital of Guangxi Medical University, Nanning, 530021, China

^3^ Information and Management College, Guangxi Medical University, Nanning, 530021, China

^4^ Pharmaceutical College, Guangxi Medical University, Nanning, 530021, China

^#^ These authors contributed equally to this article.

*** Correspondence:**

Corresponding Authors:

Ming Gao, Tel: +86-07715540585. Fax: +86-07715540585. Email address: gaoming1983125@hotmail.com. Guangxi Engineering Center in Biomedical Materials for Tissue and Organ Regeneration, The First Affiliated Hospital of Guangxi Medical University, Nanning, 530021, China.

Fangming Song, Tel: +86-07715540585. Fax: +86-07715540585. Email address: [fangmingsong109@163.com](mailto:fangmingsong109@163.com). Guangxi Engineering Center in Biomedical Materials for Tissue and Organ Regeneration, The First Affiliated Hospital of Guangxi Medical University, Nanning, 530021, China.

## Supplementary Figures


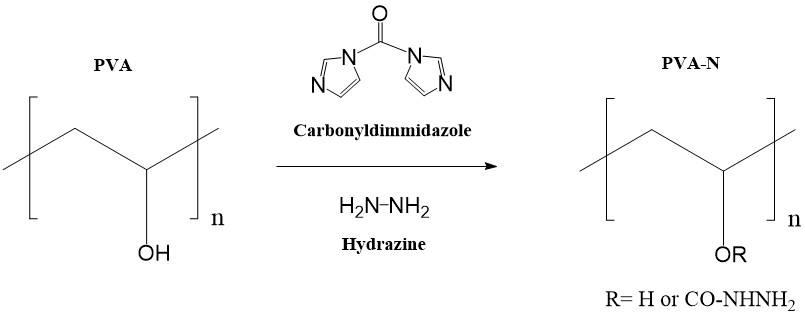


**Fig. s1**. The synthetic procedures of carbazate modified PVA (PVA-N).


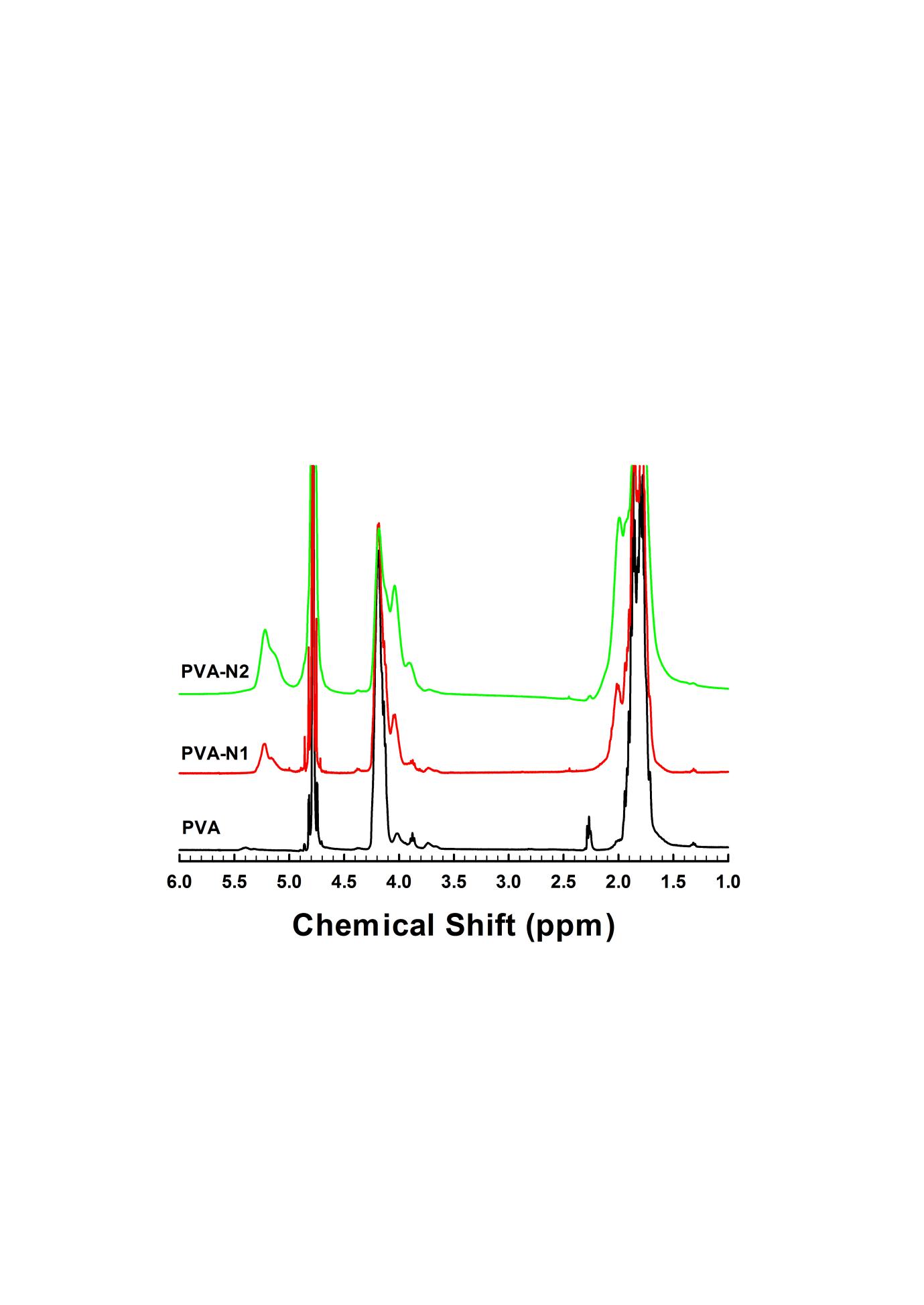


**Fig. s2**. 1H NMR results of PVA, PVA-N1 and PVA-N2 with the solvent of D2O at 40 ℃.


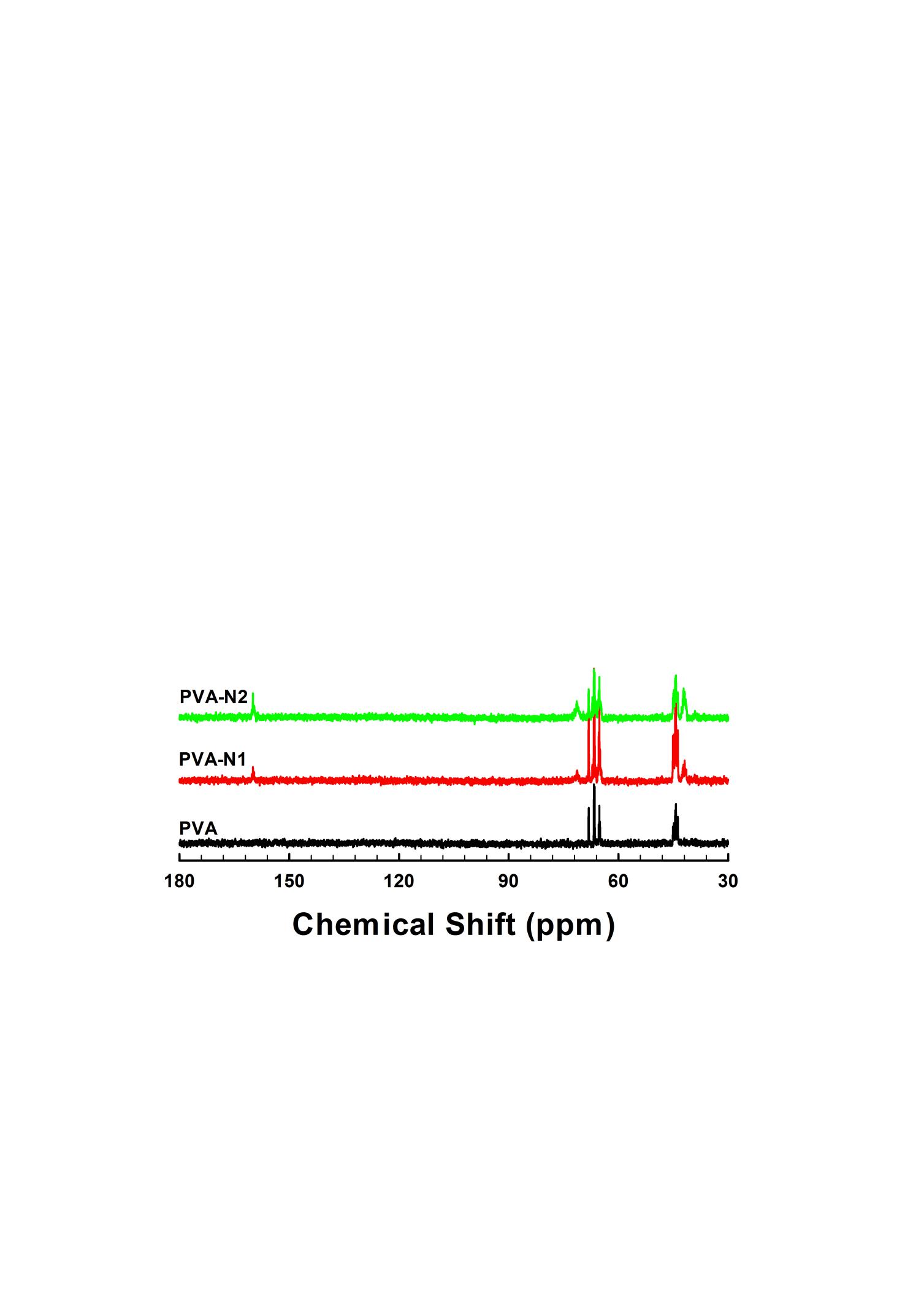


**Fig. s3**. 13C NMR results of PVA, PVA-N1 and PVA-N2 with the solvent of D_2_O at 40 ℃.


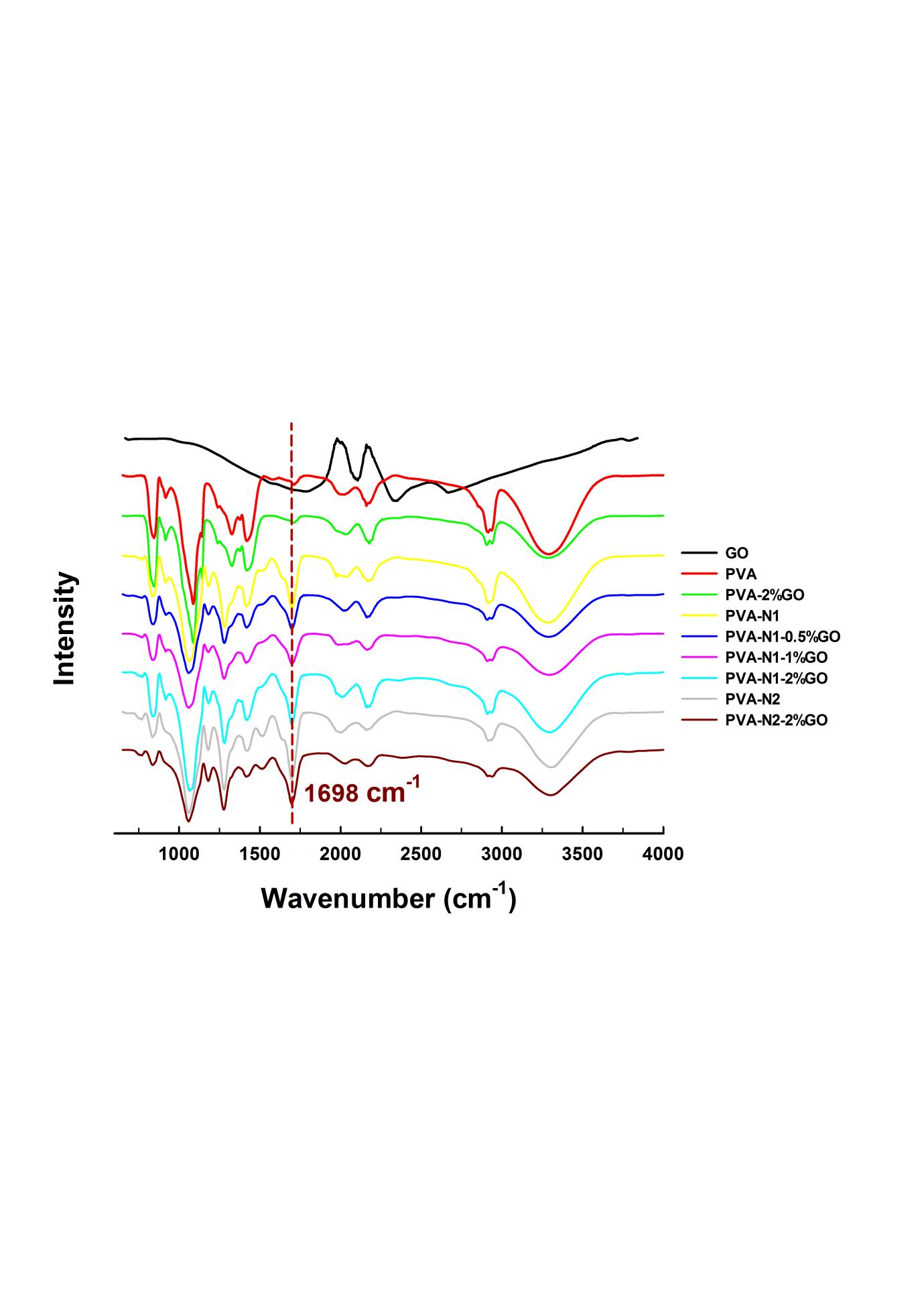


**Fig. s4**. FTIR results of GO, PVA, PVA-N and their nanocomposites with GO.


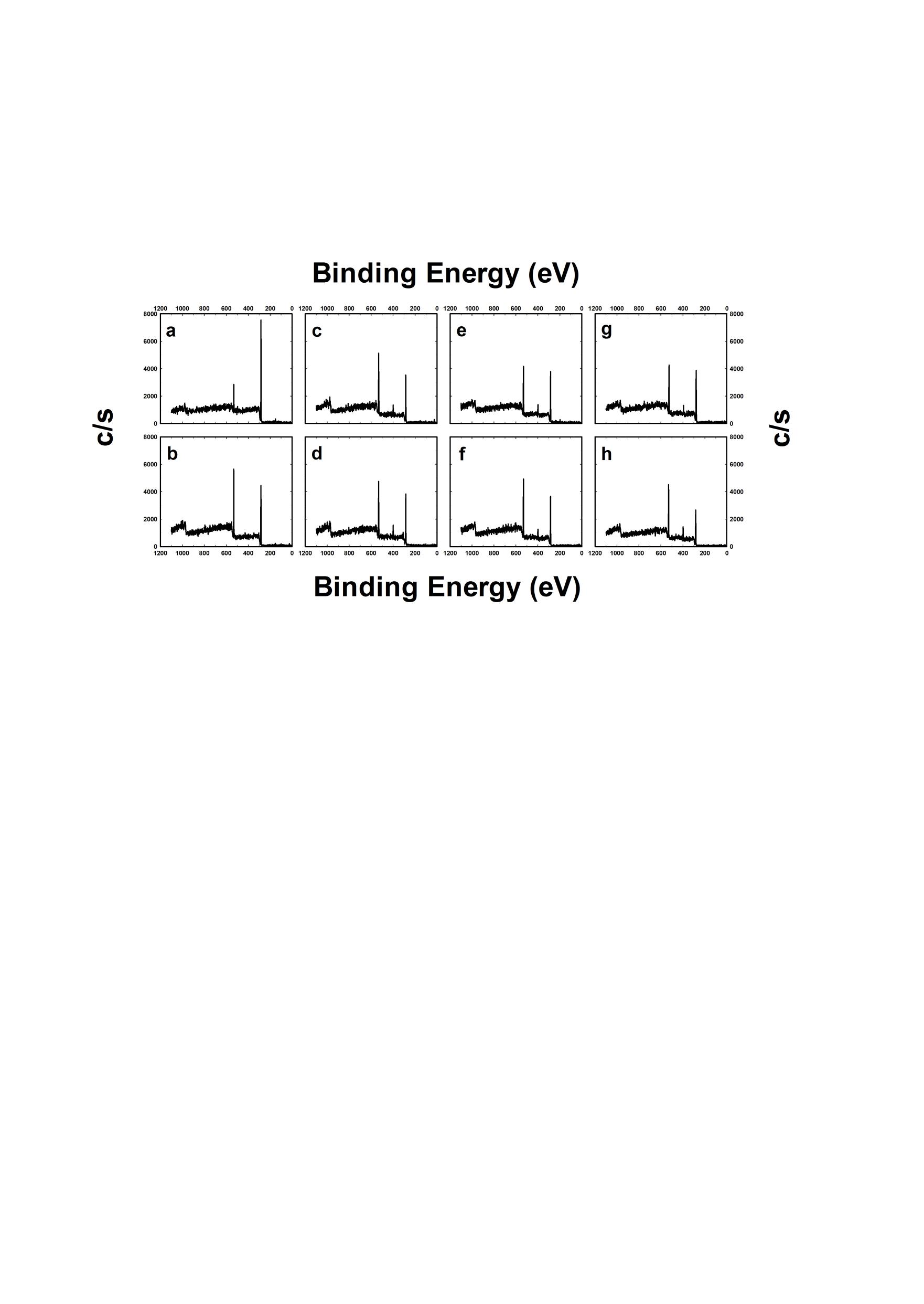


**Fig. s5**. XPS full spectrum of PVA (a), PVA-2%GO (b), PVA-N1 (c), PVA-N1-0.5%GO (d), PVA-N1-1%GO (e), PVA-N1-2%GO (f), PVA-N2 (g), and PVA-N2-2%GO (h).


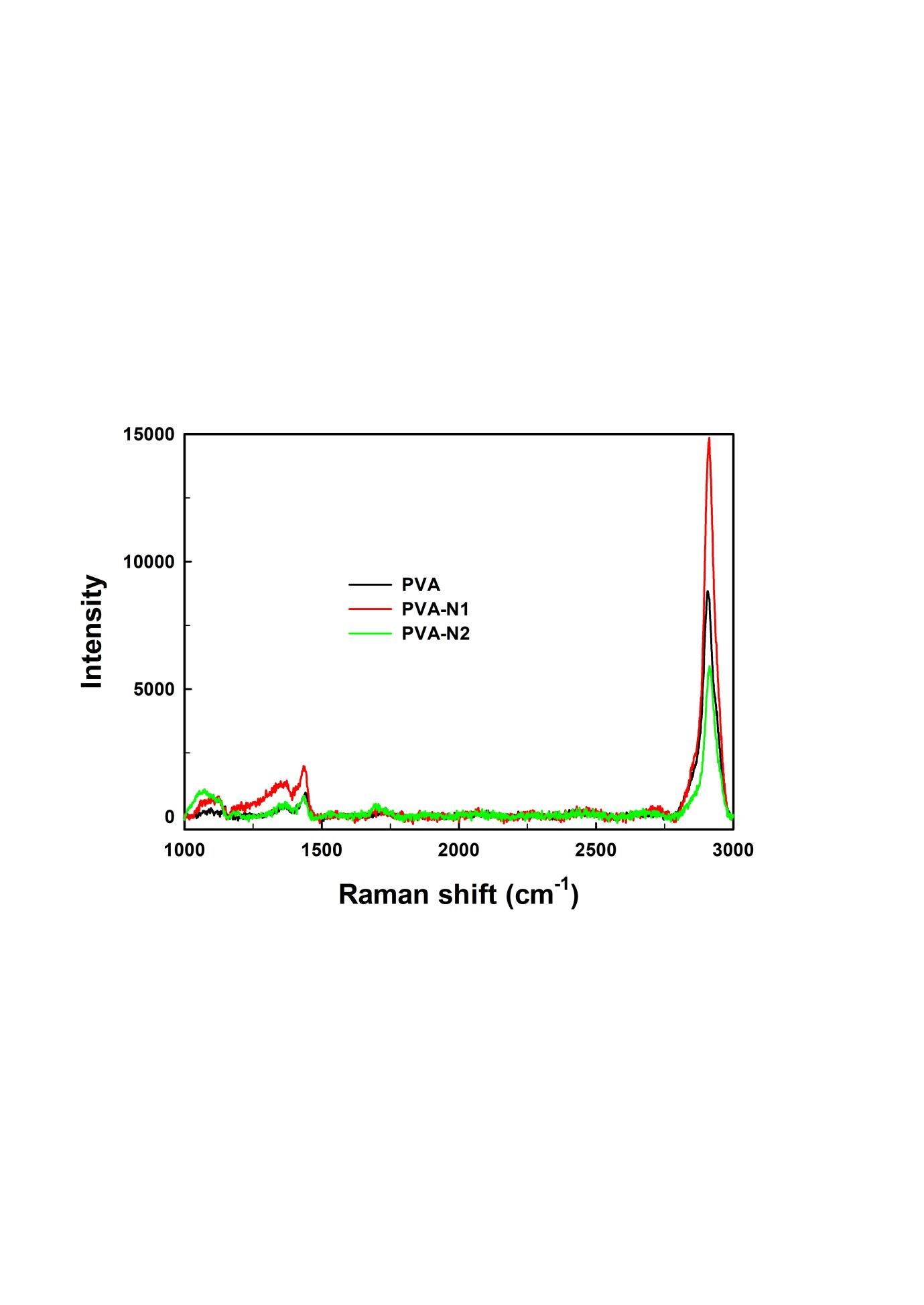


**Fig. s6**. Raman spectroscopy curves of PVA, PVA-N1 and PVA-N2.


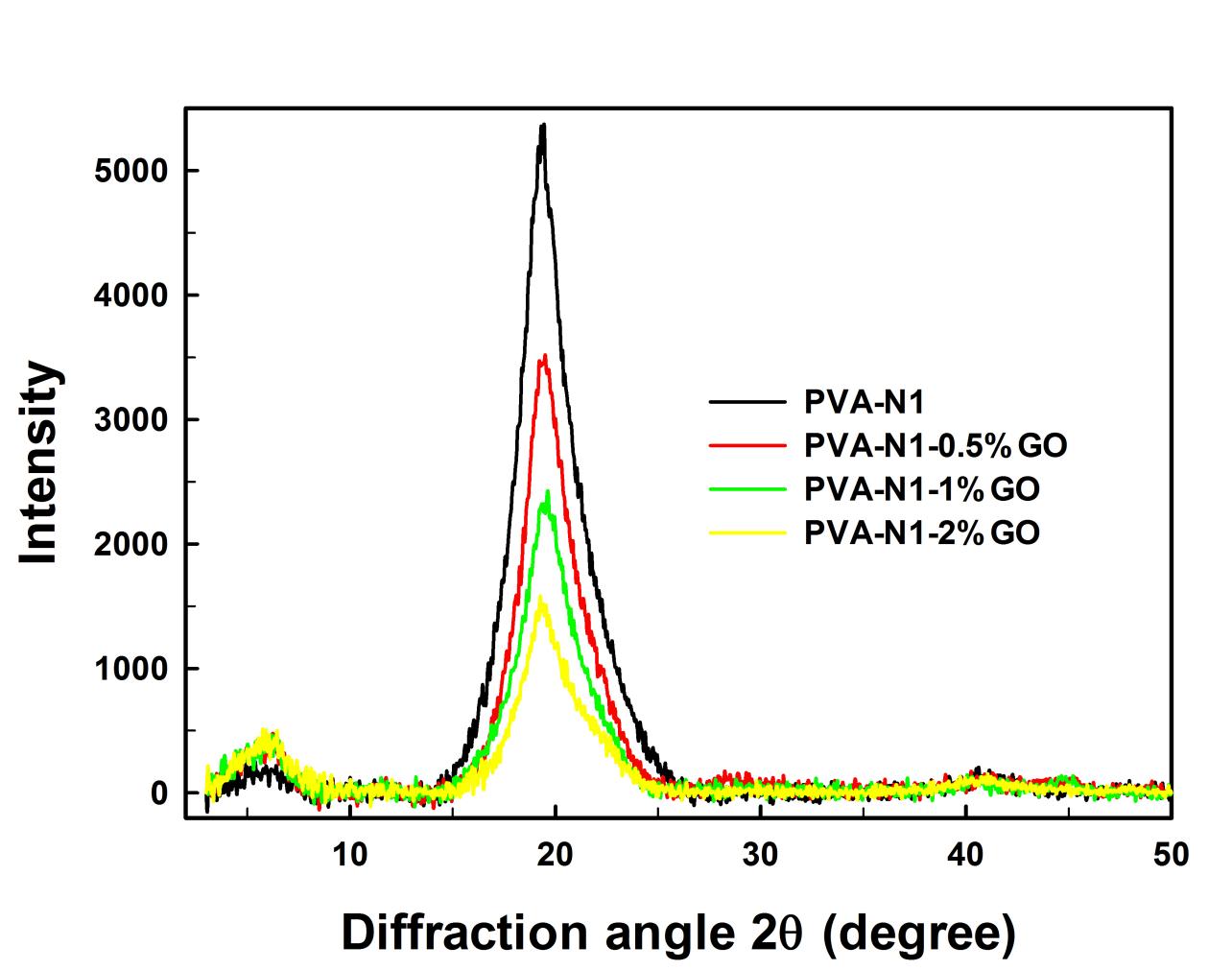


**Fig. 7**. XRD results of PVA-N1 and its nanocomposites with different wt% (0.5, 1 and 2%) of GO with the diffraction angle from 5° to 50°.


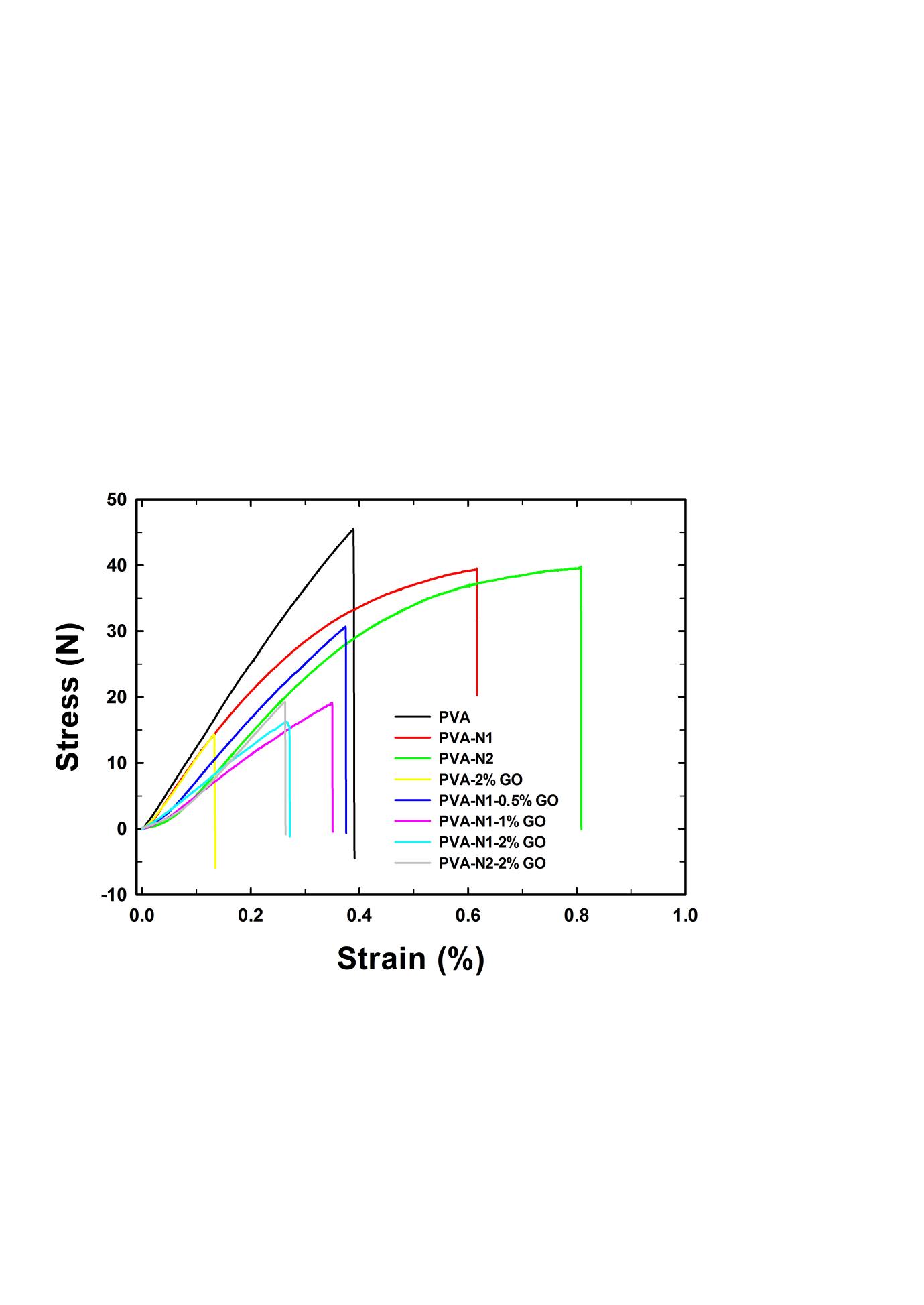


**Fig. s8**. Stress- strain curves of PVA, PVA-N and their nanocomposites with GO.


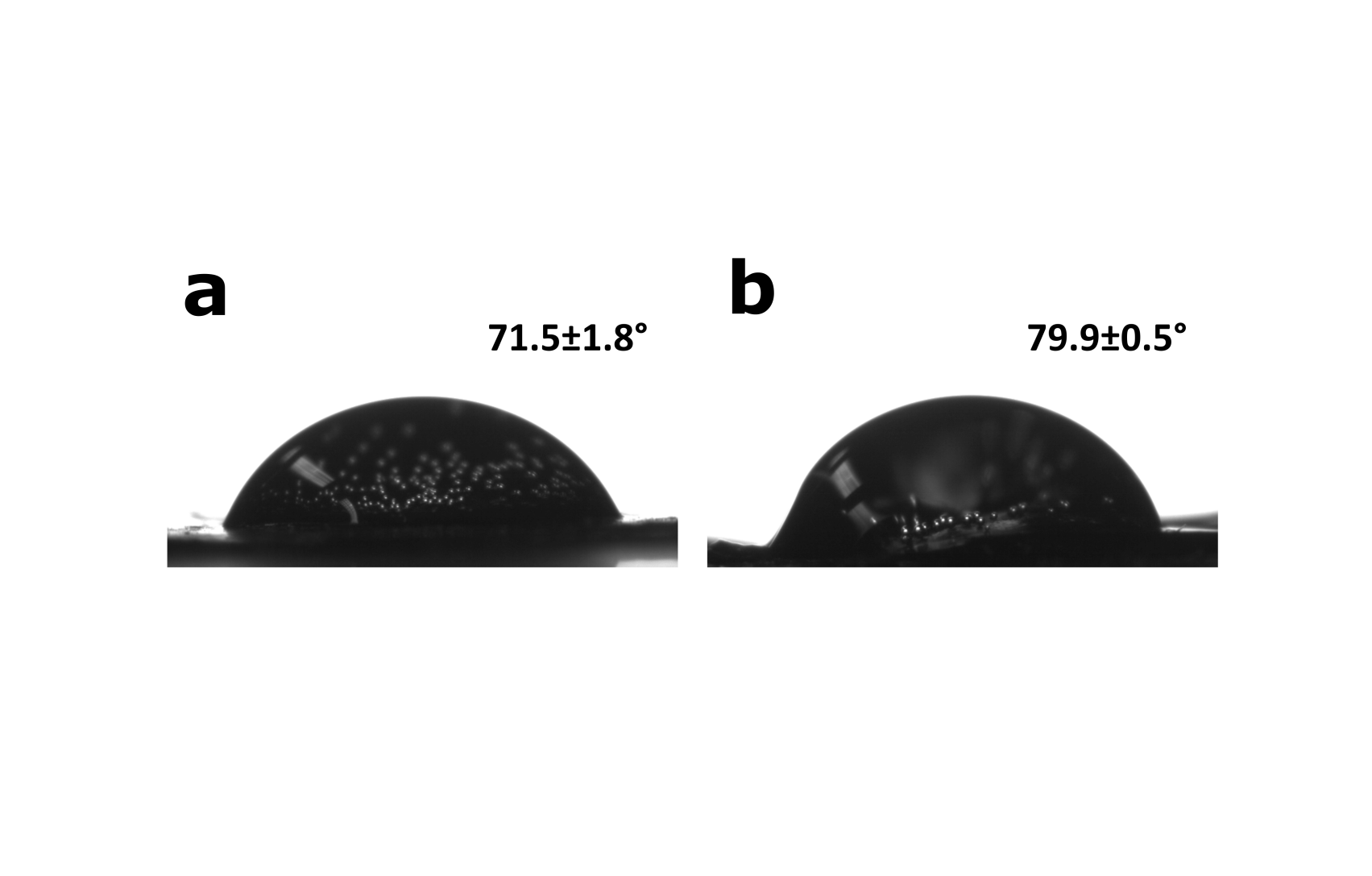


**Fig. s9**. Surface contact angle images of PVA-N1-0.5%GO (a) and PVA-N1-1%GO (b).

## Supplementary Tables

**Table s1**. The detailed recipe of PVA-N preparation

| **Name** | **PVA (g)** | **DMSO**  **(mL)** | **CDI**  **(g)** | **Hydrazine**  **(60%, mL)** | **DS (%)** |
| --- | --- | --- | --- | --- | --- |
| **PVA-N1** | 1 | 20 | 2 | 4.2 | 4 |
| **PVA-N2** | 1 | 20 | 2 | 2.1 | 7 |

**Table s2**. The detailed recipe for the preparation of nanocomposites

| **Name** | **PVA (g)** | **PVA-N1 (g)** | **PVA-N2 (g)** | **GO solution**  **(2 mg/ mL, mL)** |
| --- | --- | --- | --- | --- |
| **PVA-2%GO** | 0.1 | 0 | 0 | 1 |
| **PVA-N1-0.5%GO** | 0 | 0.1 | 0 | 0.25 |
| **PVA-N1-1%GO** | 0 | 0.1 | 0 | 0.5 |
| **PVA-N1-2%GO** | 0 | 0.1 | 0 | 1 |
| **PVA-N2-2%GO** | 0 | 0 | 0.1 | 1 |
